# Assessing Executive Function in Patients With Temporal Lobe Epilepsy Through a Game‐Based Assessment Task

**DOI:** 10.1155/bn/6284292

**Published:** 2026-04-12

**Authors:** Guang Yao, Shirui Wen, Quanji Li, Yin Liu, Beibing Li, Zhi Song, Li Feng, Ding Liu

**Affiliations:** ^1^ Department of Neurology, The Third Xiangya Hospital, Central South University, Changsha, Hunan, China, csu.edu.cn; ^2^ Department of Neurology, The Fourth Hospital of Changsha, Changsha, Hunan, China; ^3^ Department of Neurology, Xiangya Hospital, Central South University, Changsha, Hunan, China, csu.edu.cn; ^4^ National Clinical Research Center for Geriatric Disorders, Xiangya Hospital, Central South University, Changsha, Hunan, China, csu.edu.cn; ^5^ Paul G. Allen School of Computer Science and Engineering, University of Washington, Seattle, Washington, USA, washington.edu

**Keywords:** executive function, set-shifting ability, temporal lobe epilepsy, video game

## Abstract

**Objectives:**

Temporal lobe epilepsy (TLE) is often accompanied by executive function (EF) impairment, highlighting the need for accessible and quantitative assessment approaches. In this study, we applied our previously developed game‐based executive function assessment tool (EFAT) to evaluate EF performance in TLE patients.

**Methods:**

Thirty‐two TLE patients and 29 healthy controls (HCs) completed a comprehensive battery of standardized tests, including the Stroop Color‐Word Test (SCWT) and the Trail Making Test (TMT), as well as the EFAT. Metrics from the EFAT (number of touches, number of correct guesses, accuracy, and learning latency) were selected and analyzed.

**Results:**

Compared with HCs, TLE patients showed significant EF deficits, including longer completion times on TMT‐A (*t*(59) = 4.42, *p* < 0.001) and TMT‐B (*t*(59) = 3.50, *p* = 0.001), as well as poorer SCWT‐1 performance (*t*(59) = 2.22, *p* = 0.036). In the EFAT, TLE patients exhibited more touches (*t*(59) = −2.75, *p* = 0.008), fewer correct guesses (*t*(59) = −3.74, *p* < 0.001), and lower accuracy (*t*(59) = −2.65, *p* = 0.010). Game‐derived metrics were significantly correlated with TMT scores.

**Conclusions:**

EFAT detected executive deficits in TLE patients across social and nonsocial conditions and offered an objective, engaging complementary tool for EF assessment. These findings support its preliminary clinical applicability.

## 1. Introduction

Temporal lobe epilepsy (TLE) is the most common form of focal epilepsy in adults. Increasing evidence from neuropsychological and neuroimaging studies indicates that TLE patients suffer from cognitive impairment, such as deficits in social cognition and executive function (EF) [1–5]. EF is a cognitive construct that refers to a set of cognitive processes that support a goal‐directed behavior. It consists of three major core components: set shifting (also known as cognitive flexibility), inhibitory control, and working memory [6]. Notably, recent findings from an epilepsy‐centric perspective on cognitive deficits indicate that these impairments, including executive dysfunction, may precede the onset of epilepsy [7–10]. As such, measures of executive dysfunction could serve as potential biomarkers for disease development and prognostic assessment. Therefore, quantitative, objective, and sensitive metrics are needed to assess EF in TLE patients.

Traditional neuropsychological assessments applied to evaluate EF include the Stroop Color‐Word Test (SCWT), the Trail Making Test (TMT), and the Wisconsin Card Sorting Test (WCST). However, the clinical implementation of conventional paper‐and‐pencil assessments is often limited by a reduced sensitivity to subtle deficits, restricted behavioral granularity, limited repeatability, and the requirement for administration by highly trained clinical professionals. Collectively, these factors constrain refined assessment and long‐term monitoring. In addition, traditional assessments primarily focus on cognitive information processing and may insufficiently capture social interaction–related deficits associated with TLE. For these reasons, there is a clear need to develop novel tools for assessing EF in patients with TLE that are more objective, efficient, and comprehensive.

Compared with traditional neuropsychological scales, mobile video games could be especially apt for the longitudinal cognitive evaluation of TLE patients. Many cognitive batteries have been gamified, and they belong to the category of “serious games” [11]. These so‐called “serious games” are designed for beneficial purposes and could complement traditional methods [12]. At present, most video games are specifically designed for the assessment of patients with attention deficit hyperactivity disorder (ADHD). These studies typically select a video game‐based continuous performance test (CPT) or a similar go/no‐go task to measure participants′ EF [13, 14]. In addition, the diagnostic ability of video game‐based assessments compared with clinical diagnosis has been demonstrated in previous studies [14–16]. With the rapid growth in the use of mobile devices, many scientists have increasingly begun to focus their research on the use of mobile apps for preliminary disease assessment as well as progression monitoring [17]. Therefore, research in this field has favorable prospects for clinical application.

In this study, we applied our previously developed game‐based executive function assessment tool (EFAT), which incorporates both social and nonsocial stimuli, to characterize EF performance in TLE patients. Building on the need for quantitative and engaging assessment approaches, we examined whether game‐derived behavioral metrics align with traditional neuropsychological measures and whether EFAT may serve as a complementary method for identifying executive deficits in TLE. Our aim was to evaluate the clinical applicability of an objective, accessible, and user‐friendly tool to support the assessment of executive dysfunction in TLE patients.

## 2. Materials and Methods

### 2.1. Participants

Thirty‐two patients (19 females) with TLE and 29 healthy controls (17 females) were recruited from Xiangya Hospital and Third Xiangya Hospital between October 2017 and March 2018. All subjects signed an informed consent form approved by the local ethics committee. Epilepsy patients were diagnosed with TLE by at least two epilepsy experts according to the International League Against Epilepsy (ILAE) classification published in 2017. Participant inclusion criteria included (1) more than 18 years of age, (2) criteria for the classification of TLE were met, (3) normal or corrected‐to‐normal vision and normal hearing, and (4) no history of long‐term use of other medications (except antiseizure medications [ASMs]). All patients were allowed to take their routine medications. Two of them did not take any medications when included in the study. Twenty‐five of them received monotherapy. The remaining five patients were taking two or more ASMs, a combination of oxcarbazepine, valproate acid, levetiracetam, lamotrigine, and other ASMs. Those with structural abnormalities related to epilepsy in other brain regions were excluded from this research.

### 2.2. Neuropsychological Assessment

The same neuropsychologist expert administered all tests and questionnaires blinded to the clinical information. All participants completed a comprehensive battery of standardized tests.

#### 2.2.1. SCWT

The SCWT is one of the most commonly used measures to assess EF [18]. A paper version of the SCWT was used. This test consists of three phases. In the preliminary phase (SCWT‐1), the subject was asked to read aloud the words that represented the colors on the cards. In the next phase (SCWT‐2), the subject was asked to read aloud the colors of the circles on the card. In the final phase (SCWT‐3), there were 48 words that included congruent colored words (the word color was identical to the meaning of the word) and incongruent colored words (the color of the word was different from the word meaning). The time to complete the task was measured in seconds. Stroop interference effects (SIEs), which reflect the word interference and color interference in SCWT, are calculated as the duration of the SCWT‐3 test divided by that of the SCWT‐1 test. A higher SIE value indicates poorer interference resistance for the participant.

#### 2.2.2. TMT

The TMT consists of two parts: TMT‐A and TMT‐B [19]. It included 25 circles distributed on a sheet of paper. The TMT‐A was used to measure an individual′s processing speed, and it consisted of drawing lines as quickly as possible to link consecutively numbered circles (1–25). In contrast, in TMT‐B, participants were to connect circles while alternating between red and yellow circles, and each color of circle was also numbered (1–25). The TMT‐B was used to measure cognitive flexibility or switching ability. The time to complete the task was measured in seconds.

### 2.3. Game Design

The EFAT used in the present study was developed based on a digital framework originally designed by members of our research team and informed by established paradigms from the NIH Toolbox Cognition Battery (Dimensional Change Card Sort and Flanker Task), which measure core constructs including set shifting, rule learning, cognitive flexibility, and inhibitory control. This platform has been previously adapted for TLE research, demonstrating its feasibility in clinical populations [20].

The EFAT consists of three game‐based tasks. The current study focuses on the shifting game due to its relevance to cognitive domains affected in TLE. The task was implemented in Unity and deployed on a Windows laptop. It employed two categories of visual stimuli: social (front‐facing human images from the validated CUHK database) and nonsocial (fractals controlled for resolution, luminance, and complexity). The social and nonsocial versions were structurally identical.

**Figure 1:**
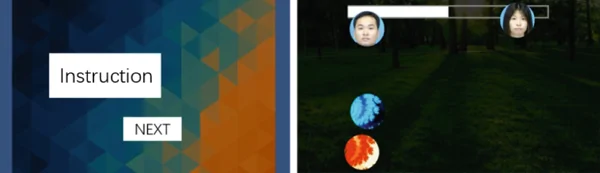
Shifting game structure and task flow. The shifting game, modeled after the Wisconsin Card Sorting Test, presented four images per trial. Participants were required to identify the target matching a hidden rule (e.g., man′s face, woman′s face, red fractal, and blue fractal). The rule changed every 10 trials without warning. At the beginning of the task, instructions were displayed on the screen to familiarize participants with the interface. In each trial, four images appeared simultaneously—either human faces or fractal patterns—and participants were required to identify the target image that matched an unspoken categorization rule. During each trial, participants could make multiple selections: Incorrect choices resulted in the immediate removal of the selected item, allowing continued exploration among the remaining stimuli, whereas selecting the correct target advanced the participant to the next trial. This design enabled detailed extraction of behavioral indices such as number of touches, correct guesses, accuracy, and learning latency, reflecting participants′ strategy use, rule acquisition, and set‐shifting ability.

The shifting game, modeled after the WCST, presented four images per trial. Participants were required to identify the target matching a hidden rule (e.g., man′s face, woman′s face, red fractal, and blue fractal) (Figure 1). The rule changed every 10 trials without warning. Participants could make multiple selections per trial; incorrect choices led to stimulus removal, allowing exploration until the correct answer was selected. The task began with 12 practice trials (excluded from analysis), and instructions were provided via on‐screen text and auditory guidance. Immediate auditory feedback (“yes”/“no”) was given for each selection.

The following behavioral indices were automatically recorded:

1. Number of touches: total clicks per trial, reflecting search effort and decision strategy.
2. Correct guesses: number of trials with a correct first attempt, reflecting rule acquisition.
3. Accuracy: proportion of correct guesses relative to total trials.
4. Learning latency: number of trials required to identify the rule within each block, reflecting set shifting and cognitive flexibility.

This structure allows for a fine‐grained assessment of EFs impaired in TLE.

### 2.4. Blinding Procedures

To minimize procedural and analytic bias, different components of the study followed distinct blinding practices. Behavioral task administration and scoring were performed by trained staff who were not blinded to group assignment. In contrast, neuropsychological testing was conducted by an experienced neuropsychologist who was blinded to clinical information. All behavioral data analyses were subsequently carried out by researchers blinded to participants′ clinical details.

### 2.5. Statistical Analyses

Categorical variables were analyzed using chi‐square tests, and continuous variables were compared between TLE patients and healthy controls using independent samples *t*‐tests to examine group differences in participant characteristics. The normality of game‐derived metrics was assessed using the Shapiro–Wilk test prior to conducting parametric analyses. As the game metrics met normality assumptions, group comparisons were performed using independent samples *t*‐tests. All tests were two‐tailed, and a *p* value of < 0.05 was considered statistically significant unless otherwise specified. When multiple comparisons were conducted, Bonferroni correction was applied to adjust the significance threshold accordingly. Specifically, chi‐square tests were used to assess intergroup differences in proportions (including sex, etiology, number of medications, course of epilepsy, and laterality of epilepsy) and to identify univariable risk factors for executive impairment in TLE patients, with Bonferroni correction applied where multiple tests were performed. Pearson correlation coefficients were used to examine associations between variables of interest, with Bonferroni correction applied to control for multiple comparisons. Statistical analyses were performed using SPSS (Version 24.0, SPSS Inc., Chicago, Illinois, United States) and R (Version 4.0.5).

## 3. Results

### 3.1. Demographics

Thirty‐two patients with TLE and 29 healthy controls were included in the study. Demographic characteristics are summarized in Table 1. There were no significant between‐group differences in sex (*χ* ^2^ = 0.04, *p* = 0.85), age (*t*(59) = 1.10, *p* = 0.25), or years of education (*t*(59) = 0.51, *p* = 0.12). Detailed demographic and clinical characteristics are shown in Table 1. Among TLE patients, no significant differences were observed across clinical variables, including duration of epilepsy, seizure laterality, number of antiepileptic medications, and etiology.

**Table 1:** Characteristics of participants. ASMs, antiseizure medications.

	TLE patients (*n* = 32)	Healthy controls (*n* = 29)	*F* (*t*, *x* ^2^, *Z*)	*p*
Gender (female/male)	13/19	12/17	0.04	0.85
Age (mean ± SD)	30.75 ± 9.09	33.72 ± 10.72	1.10	0.25
Education (years)	12.62 ± 3.25	14.03 ± 4.00	0.51	0.12
Course of epilepsy (years)	10.32 ± 9.10	/	/	/
ASMs (0–1/≥ 2 [i.e., polytherapy])	27/5	/	/	/
Laterality (left/right)	15/17	/	/	/
Etiology (structural/unknown)	18/14	/	/	/

### 3.2. Neuropsychological Assessment

Compared with healthy controls, TLE patients required significantly longer time to complete EF tasks, including TMT‐A (*t*(59) = 4.42, *p* < 0.001, *d* = 0.97), TMT‐B (*t*(59) = 3.50, *p* = 0.001, *d* = 0.77), and SCWT‐1 (*t*(59) = 2.22, *p* = 0.036, *d* = 0.57) (Figure 2). These results indicate moderate‐to‐large impairments in EF among TLE patients. No group differences were found in SCWT‐2, SCWT‐3, or SIE performance (all *p* > 0.05).

Figure 2Time spent by patients and controls in (a) TMT‐A, (b) TMT‐B, and (c) SCWT‐1. Statistical significance for each task between groups is indicated by asterisks ( ^∗^ *p* < 0.05,  ^∗∗^ *p* < 0.01, and  ^∗∗∗^ *p* < 0.001).

**Figure:**
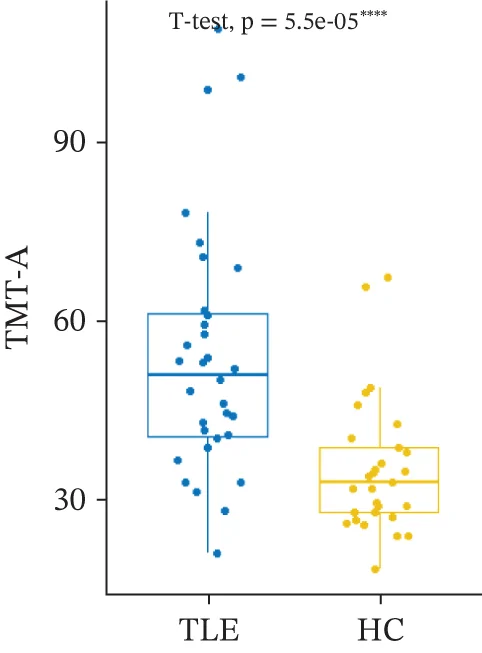
(a)

**Figure:**
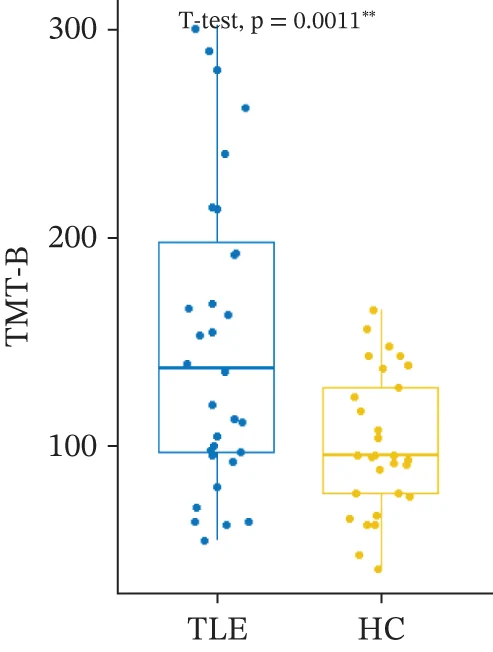
(b)

**Figure:**
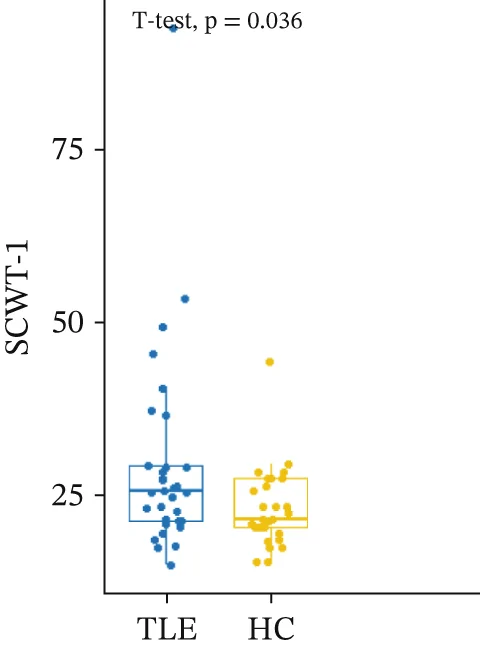
(c)

### 3.3. Shifting Game

Group‐level performance is presented in Table 2, with individual data illustrated in Figures 3, 4, 5, and 6. TLE patients made significantly more touches than healthy controls across the whole task (*t*(59) = −2.75, *p* = 0.008, *d* = −0.71), in social trials (*t*(59) = −2.29, *p* = 0.026, *d* = −0.59), and in nonsocial trials (*t*(59) = −2.92, *p* = 0.005, *d* = −0.75) (Figure 3).

**Table 2:** Patient and control group neuropsychological assessment and shifting game metrics.

	TLE patients (*n* = 32)	Healthy controls (*n* = 29)	*t*	*p*	*d*
TMT‐A	54.11 ± 20.91	35.28 ± 11.43	4.418	< 0.001	0.974
TMT‐B	153.25 ± 75.48	101.68 ± 33.52	3.502	0.001	0.772
SCWT‐1	74.73 ± 23.92	61.76 ± 20.79	2.221	0.036	0.569
Shifting game					
Touches—Whole	35.78 ± 18.74	46.66 ± 11.56	−2.754	0.008	−0.706
Touches—Social	20.16 ± 9.59	24.76 ± 5.86	−2.286	0.026	−0.586
Touches—Nonsocial	15.63 ± 9.81	21.90 ± 6.80	−2.924	0.005	−0.750
Correct guesses—Whole	20.72 ± 12.51	31.83 ± 10.44	−3.743	< 0.001	−0.960
Correct guesses—Social	12.75 ± 5.62	17.14 ± 4.24	−3.460	0.001	−0.887
Correct guesses—Nonsocial	7.97 ± 7.17	14.69 ± 6.58	−3.802	< 0.001	−0.975
Accuracy—Whole	0.57 ± 0.16	0.68 ± 0.16	−2.651	0.010	−0.680
Accuracy—Social	0.65 ± 0.14	0.70 ± 0.15	−1.370	0.176	−0.351
Accuracy—Nonsocial	0.42 ± 0.30	0.63 ± 0.23	−2.959	0.004	−0.759
Learning latency—Whole	4.82 ± 2.69	3.66 ± 2.62	1.693	0.096	0.434
Learning latency—Social	5.46 ± 3.08	3.55 ± 2.88	2.503	0.015	0.642
Learning latency—Nonsocial	4.27 ± 3.32	3.68 ± 3.11	0.663	0.510	0.170

*Note:* TMT‐A, the time needed to complete the TMT‐A; TMT‐B, the time needed to complete the TMT‐B; SCWT‐1, the time needed to complete the first part of the Stroop Color‐Word Test. Touches—whole, number of touches made across all trials of the shifting game. Touches—social, number of touches recorded during trials presenting social stimuli. Touches—nonsocial, number of touches recorded during trials presenting nonsocial stimuli. Correct guesses—whole, number of correct guesses made across all trials of the shifting game. Correct guesses—social, number of correct guesses recorded during trials presenting social stimuli. Correct guesses—nonsocial, number of correct guesses recorded during trials presenting nonsocial stimuli. Accuracy—whole, accuracy calculated across all trials of the shifting game. Accuracy—social, accuracy calculated during trials presenting social stimuli. Accuracy—nonsocial, accuracy calculated during trials presenting nonsocial stimuli. Learning latency—whole, total number of trials required to infer the hidden rules across the shifting game. Learning latency—social, number of trials required to infer the hidden rules during social‐stimulus trials. Learning latency—nonsocial, number of trials required to infer the hidden rules during nonsocial‐stimulus trials.

Figure 3(a) Number of touches in the shifting game, (b) number of touches in the shifting game with social stimuli, and (c) number of touches in the shifting game with nonsocial stimuli.

**Figure:**
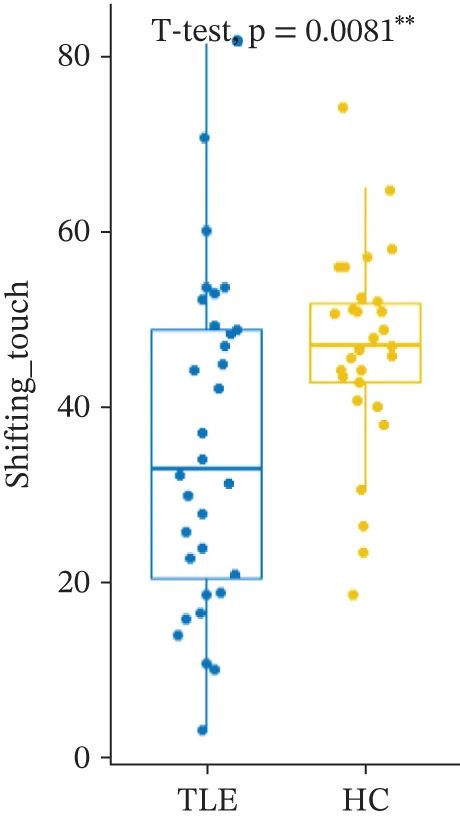
(a)

**Figure:**
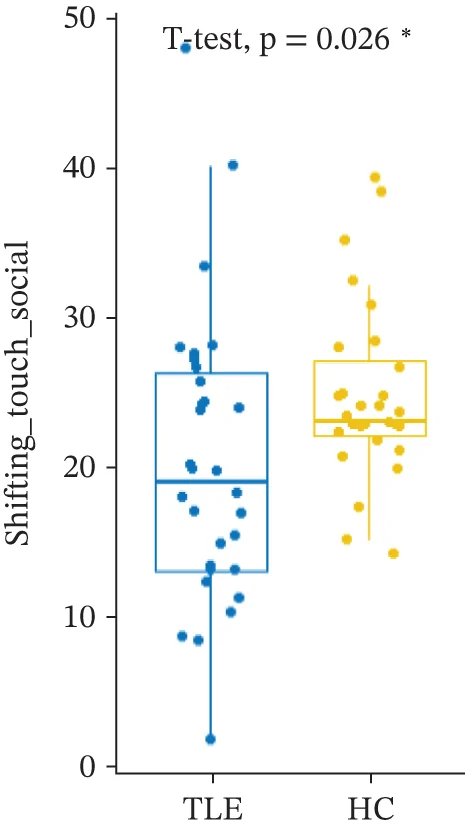
(b)

**Figure:**
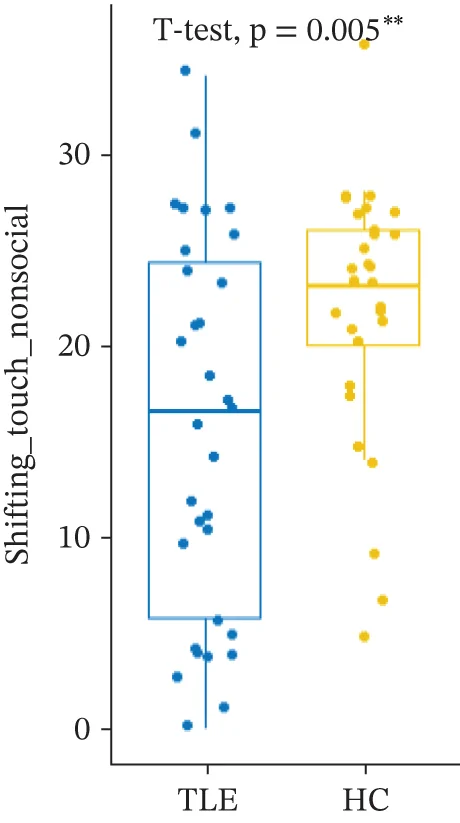
(c)

Figure 4(a) Number of correct guesses in the shifting game, (b) number of correct guesses in the shifting game with social stimuli, and (c) number of correct guesses in the shifting game with nonsocial stimuli.

**Figure:**
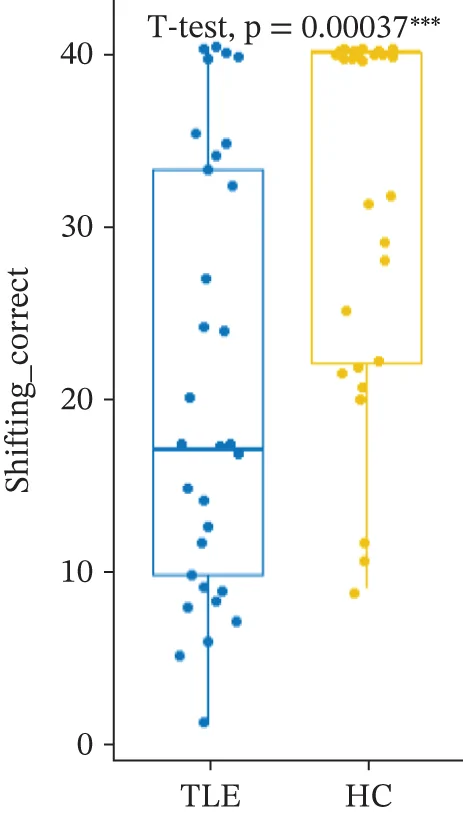
(a)

**Figure:**
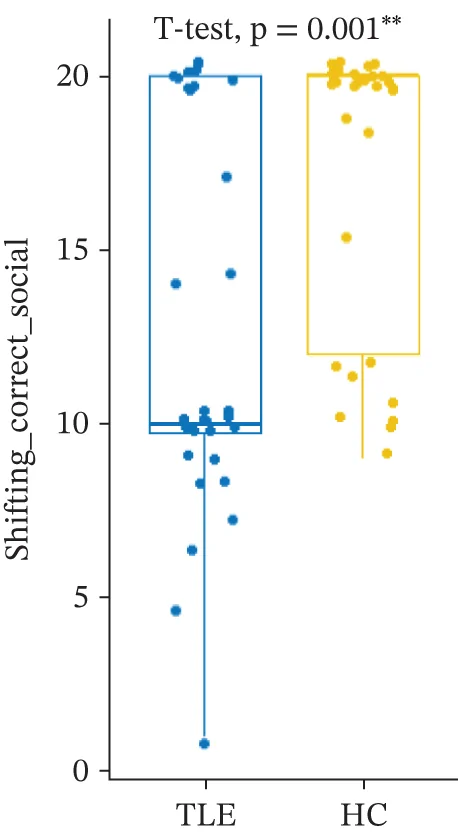
(b)

**Figure:**
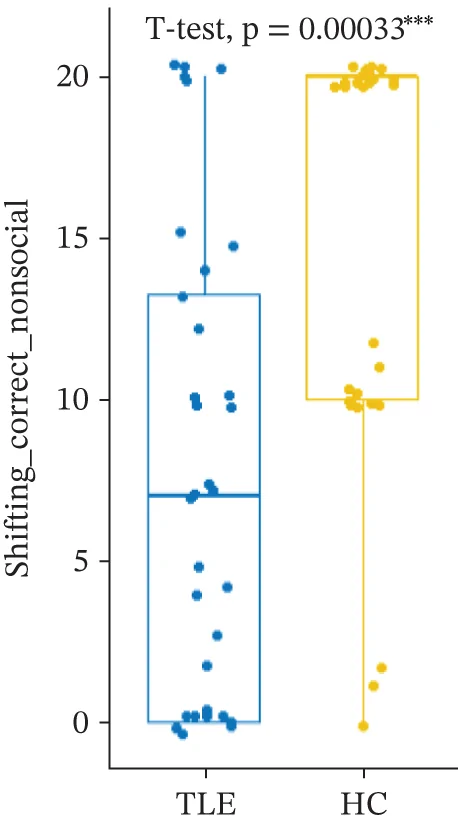
(c)

Figure 5(a) The accuracy in the shifting game, (b) the accuracy in the shifting game with social stimuli, and (c) the accuracy in the shifting game with nonsocial stimuli.

**Figure:**
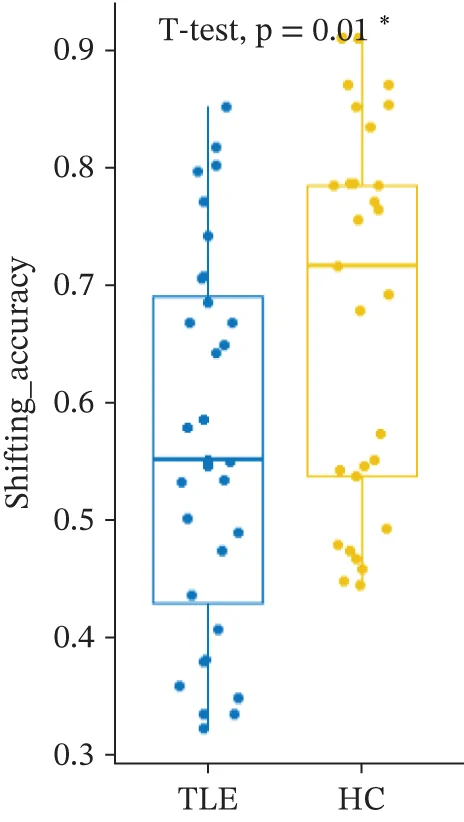
(a)

**Figure:**
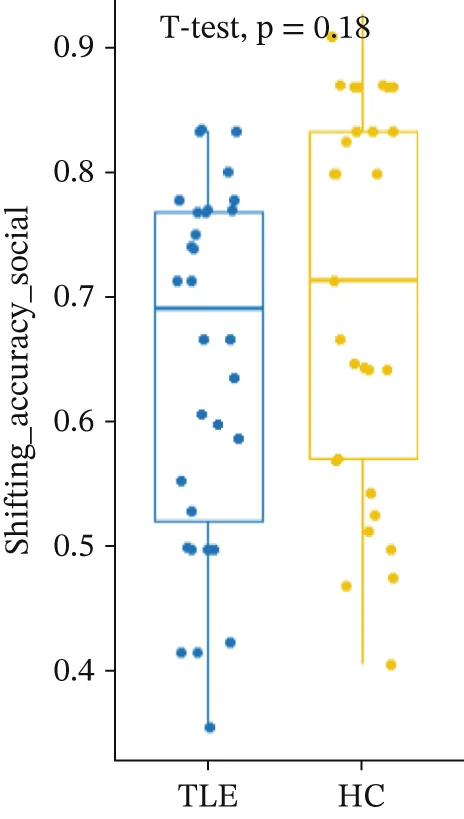
(b)

**Figure:**
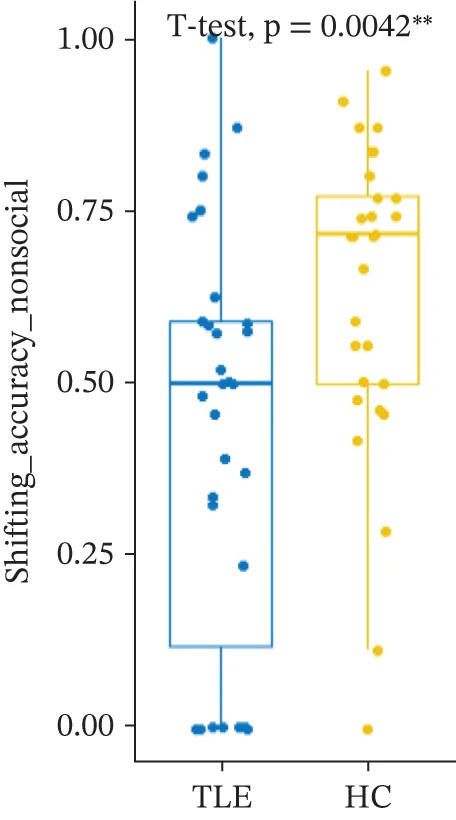
(c)

Figure 6(a) The learning latency in the shifting game, (b) the learning latency in the shifting game with social stimuli, and (c) the learning latency in the shifting game with nonsocial stimuli.

**Figure:**
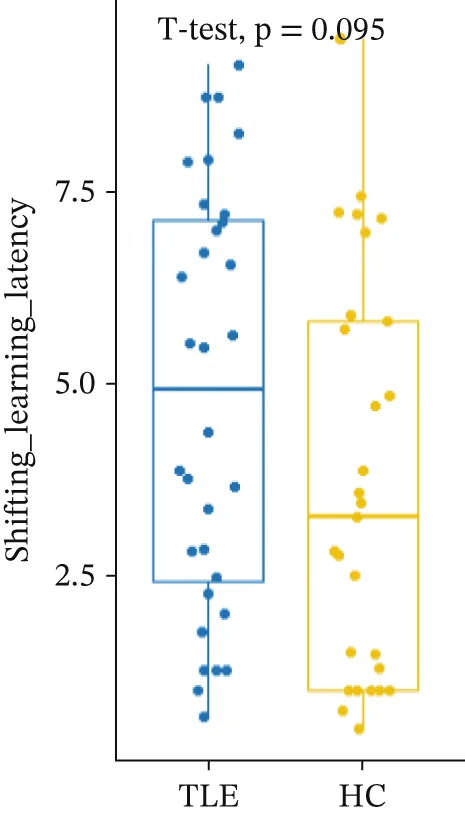
(a)

**Figure:**
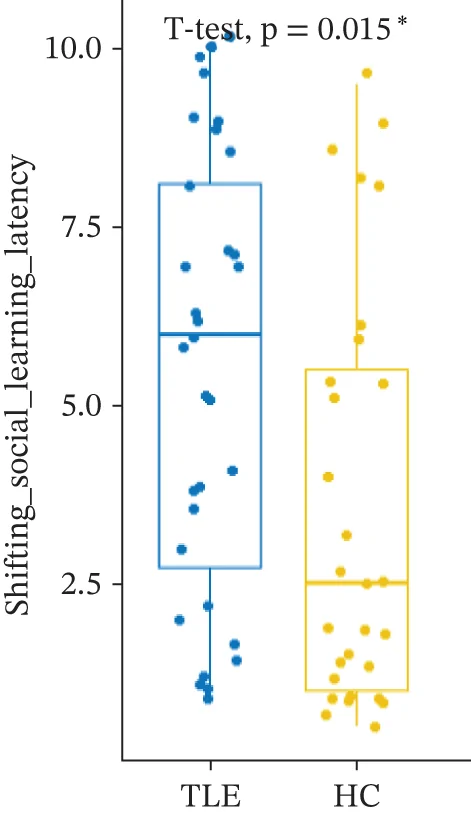
(b)

**Figure:**
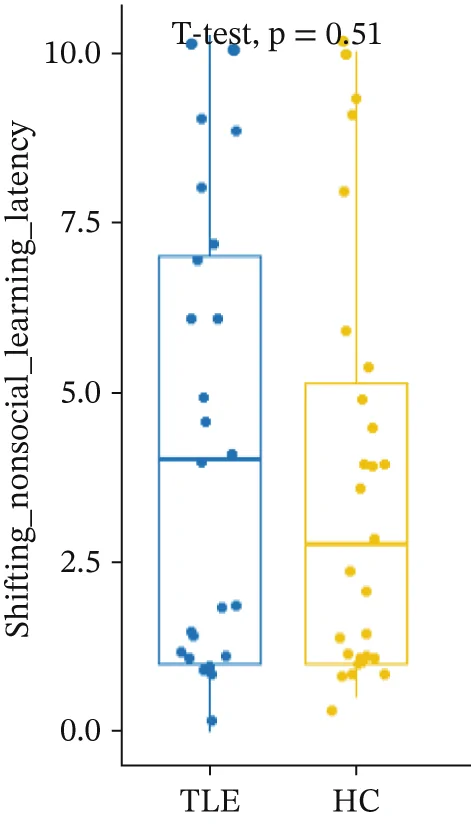
(c)

The number of correct guesses was also significantly lower in TLE patients for the whole task (*t*(59) = –3.74, *p* < 0.001, *d* = –0.96), social trials (*t*(59) = –3.46, *p* = 0.001, *d* = –0.89), and nonsocial trials (*t*(59) = –3.80, *p* < 0.001, *d* = –0.97) (Figure 4).

Similarly, task accuracy was reduced in TLE patients for the whole task (*t*(59) = –2.65, *p* = 0.010, *d* = –0.68) and in nonsocial trials (*t*(59) = –2.96, *p* = 0.004, *d* = –0.76), whereas the social trials did not show significant group differences (*p* = 0.176) (Figure 5).

Learning latency was significantly longer in TLE patients during social trials (*t*(59) = 2.50, *p* = 0.015, *d* = 0.64) but not in nonsocial trials (*t*(59) = 0.66, *p* = 0.510, *d* = 0.17) (Figure 6). Overall, the effect sizes indicate moderate‐to‐large impairments in cognitive flexibility and rule learning across most shifting game measures.

### 3.4. Correlation Analysis

**Figure 7:**
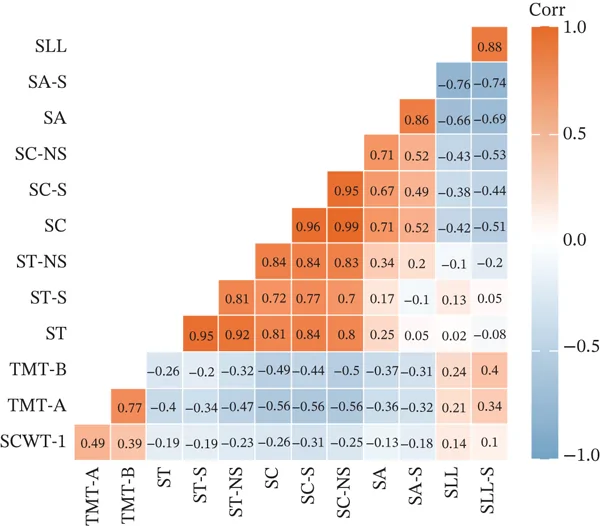
ST, number of touches in the shifting game; ST‐S, number of touches in the shifting game with social stimuli; ST‐NS, number of touches in the shifting game with nonsocial stimuli; SC, number of correct guesses in the shifting game; SC‐S, number of correct guesses in the shifting game with social stimuli; SC‐NS, number of correct guesses in the shifting game with nonsocial stimuli; SA, the accuracy rate in the shifting game; SA‐S, the accuracy rate in the shifting game with social stimuli; SLL, the learning latency in the shifting game; SLL‐S, the learning latency in the shifting game with social stimuli.

Correlation analysis results are reported in the following text and visualized in the correlation matrix (Figure 7). In the correlation analysis, the number of correct guesses showed a significant negative association with executive performance on both TMT‐A and TMT‐B across all task conditions. Specifically, fewer correct guesses were associated with longer completion times on TMT‐A during the whole task (*r* (59) = −0.56, *p* < 0.01), social trials (*r* (59) = −0.56, *p* < 0.01), and nonsocial trials (*r* (59) = −0.56, *p* < 0.01), and similar relationships were observed for TMT‐B during the whole task (*r* (59) = −0.49, *p* < 0.01), social trials (*r* (59) = −0.44, *p* < 0.01), and nonsocial trials (*r* (59) = −0.5, *p* < 0.01). Learning latency also showed significant positive correlations with TMT performance, indicating that participants who required more trials to acquire the rule tended to exhibit poorer EF. This pattern was evident for both TMT‐A (whole task *r* (59) = 0.21, *p* < 0.05; social trials *r* (59) = 0.34, *p* < 0.05) and TMT‐B (whole task *r* (59) = 0.24, *p* < 0.01; social trials *r* (59) = 0.40, *p* < 0.01). These findings consistently demonstrate that poorer executive functioning is associated with reduced guessing accuracy and slower rule acquisition in the shifting game, further underscoring the close link between attentional control, cognitive flexibility, and performance in dynamic decision‐making tasks.

## 4. Discussion

The present study evaluated the applicability of our previously developed game‐based EFAT in patients with TLE and demonstrated its capacity to detect executive dysfunction in this population. Compared with healthy controls, TLE patients exhibited poorer performance across multiple EFAT indices, including reduced touches, fewer correct responses, and lower accuracy in both the social and nonsocial conditions of the shifting game. Importantly, EFAT performance patterns were consistent with those observed on conventional neuropsychological tasks, and EFAT indices showed significant correlations with TMT scores, supporting the tool′s clinical relevance for assessing EF in TLE.

Our findings align with extensive prior evidence indicating that TLE is associated with broad executive impairments, including deficits in set shifting, inhibition, and strategic processing [2, 21–24]. Social cognition difficulties have also been reported in TLE [5, 25], and recent studies have further characterized mesial TLE as presenting a distinct EF impairment profile [26]. In line with these observations, the present study demonstrated that TLE patients showed poorer shifting performance than healthy controls in both social and nonsocial domains. Interestingly, TLE patients exhibited a longer learning latency and required more time to identify hidden rules when social stimuli were presented, whereas group differences were not significant in the nonsocial condition. This pattern may reflect social cognitive deficits in TLE, which could increase avoidance of social stimuli and thereby contribute to poorer executive performance during socially relevant trials [27–29]. Although the mechanisms underlying set‐shifting impairment in TLE remain unclear, its pathogenesis is likely multifactorial. Previous studies suggest a close association with frontal lobe dysfunction, particularly involving the left frontal regions [3]. For example, Leyden et al. reported that reduced integrity of fiber tracts connecting the frontal lobes (including the uncinate fasciculus, arcuate fasciculus, dorsal cingulum, and inferior fronto‐occipital fasciculus) may contribute to impaired set‐shifting ability [30]. In addition, prefrontal hypometabolism, compromised inferior frontal lobe function, and reduced connectivity between the frontal cortex and the caudate nucleus have been implicated in set‐shifting deficits in TLE [31–33].

Beyond replicating established neuropsychological findings, the present study highlights several advantages of EFAT as a complementary tool for assessing EF. EFAT metrics were significantly correlated with TMT scores among TLE patients, indicating that EFAT captures aspects of executive performance that overlap with those assessed by conventional neuropsychological measures. While traditional assessments such as the TMT and SCWT remain clinically valuable, their application is limited by reduced ecological validity, lower sensitivity to subtle impairments, and restricted characterization of higher order behavioral strategies. EFAT, in contrast, provides a more interactive and engaging assessment environment and records more fine‐grained behavioral metrics—including touch patterns, guessing strategies, search sequences, and responses to errors—that are not accessible through standard paper‐and‐pencil instruments. These richer behavioral traces may enhance the sensitivity of EF assessment, particularly for detecting mild impairments.

In addition to its advantages over traditional neuropsychological tasks, EFAT differs in meaningful ways from existing game‐based EF assessments. Several cognitive batteries have been adapted into gamified formats, with the video game–based WCST being one of the most widely used. Although the WCST is a classic paradigm for assessing rule shifting and cognitive flexibility, its digital versions often retain a key limitation of the original task: Incorrect responses typically terminate the trial immediately, offering limited opportunity to observe how individuals search for and update hypotheses about the hidden rule [34]. In contrast, the shifting game in EFAT was deliberately designed to allow continuous exploration. Following an incorrect response, the selected item disappears from the screen, enabling participants to continue testing remaining options until the correct choice is identified. This iterative feedback structure allows more fine‐grained characterization of rule discovery, strategy adjustment, and adaptive behavior—processes that may be especially informative for detecting mild or early‐stage executive dysfunction. Moreover, EFAT′s user‐friendly interface and ease of administration support participant engagement and may extend its applicability to clinical and home‐based settings. Its culture‐neutral EF constructs, together with stimuli and interface designs that are readily adaptable, further support its potential for cross‐cultural use. While the portability of EFAT suggests promise for future remote assessment, such applications will require rigorous validation through longitudinal and test–retest studies.

To date, the EFAT has been applied as an exploratory digital assessment of executive functioning across several populations. Previous studies have demonstrated its feasibility and sensitivity to executive performance patterns in healthy individuals and clinical groups, including patients with TLE using the memory‐related component. EFAT metrics have also shown associations with established neuropsychological measures. However, comprehensive psychometric validation—such as formal assessments of construct validity, reliability, and temporal stability—has not yet been systematically established. Accordingly, the present study should be interpreted as an exploratory application of the EFAT shifting task in TLE rather than a confirmatory psychometric validation.

This study has several limitations that should be considered when interpreting the findings. First, although EFAT is grounded in established paradigms and has demonstrated feasibility in prior research, it has not yet undergone full psychometric validation in epilepsy populations. Second, the single–time‐point, cross‐sectional design precludes examination of practice effects and temporal stability, and although data analysis was blinded, the lack of blinding during task administration may introduce some degree of procedural bias. Third, the relatively small sample size may limit statistical power and the generalizability of the findings to the broader TLE population; accordingly, the reported risk and correlation analyses are intended to describe associative relationships and should be interpreted with appropriate caution. Finally, clinical confounding factors, particularly the known cognitive effects of ASMs, represent an inherent and unavoidable source of variability in this patient group. Future studies employing larger samples, longitudinal designs, and rigorous psychometric evaluation of EFAT are warranted to confirm and extend these findings.

## 5. Conclusion

In this study, we applied our previously developed game‐based EFAT to explore and quantify EF in patients with TLE, differentiating between social and nonsocial performance to better understand overall patterns of cognitive deficits. Our findings revealed that TLE patients with executive deficits exhibited fewer touches, fewer correct guesses, and lower accuracies. While acknowledging the limitations of this study, we have introduced a quantitative, objective, and user‐friendly method for monitoring EF in TLE patients, thereby underscoring the need for continued research.

## Author Contributions

G.Y. and S.W. drafted the complete manuscript and made figures. G.Y., Q.L., and Y.L. contributed to the acquisition of data. S.W. and G.Y. contributed to the analysis of data. S.W. performed the systematic literature search. B.L. designed the executive function assessment game. D.L. and Z.S. revised the manuscript for important intellectual content. D.L. and L.F. approved the final draft. G.Y. and S.W. contributed equally to this work and share first authorship.

## Funding

This study was supported by the National Key Research and Development Program of China (10.13039/501100012166, 2022YFC2503804), the National Natural Science Foundation of China (10.13039/501100001809, 81801295 and 82271503), and the Wisdom Accumulation and Talent Cultivation Project of the Third Xiangya Hospital of Central South University (YX202205).

## Ethics Statement

This study was approved by the ethics committee of the Third Xiangya Hospital of Central South University. All data were confidential. Participants provided informed consent before participating in the study and were allowed to withdraw from the study at any phase.

## Conflicts of Interest

The authors declare no conflicts of interest.

## Data Availability

The datasets used and analyzed during the current study are available from the corresponding author on reasonable request.
